# Transmission-blocking strategies: the roadmap from laboratory bench to the community

**DOI:** 10.1186/s12936-016-1163-3

**Published:** 2016-02-18

**Authors:** Daniel Gonçalves, Patrick Hunziker

**Affiliations:** CLINAM Foundation for Nanomedicine, University of Basel, Basel, Switzerland

**Keywords:** Transmission blocking strategies, Refractory mosquitoes, Gametocytocidal drugs, Malaria vaccine

## Abstract

Malaria remains one of the most prevalent tropical and infectious diseases in the world, with an estimated more than 200 million clinical cases every year. In recent years, the mosquito stages of the parasite life cycle have received renewed attention with some progress being made in the development of transmission-blocking strategies. From gametocytes to late ookinetes, some attractive antigenic targets have been found and tested in order to develop a transmission blocking vaccine, and drugs are being currently screened for gametocytocidal activity, and also some new and less conventional approaches are drawing increased attention, such as genetically modified and fungus-infected mosquitoes that become refractory to *Plasmodium* infection. In this review some of those strategies focusing on the progress made so far will be summarized, but also, the challenges that come from the translation of early promising benchwork resulting in successful applications in the field. To do this, the available literature will be screened and all the pieces of the puzzle must be combined: from molecular biology to epidemiologic and clinical data.

## Background

### Why block transmission?

Malaria remains one of the most prevalent tropical and infectious diseases in the world, both, in terms of morbidity and mortality, with around 200 million cases estimated in 2013 alone [1]. Four different species consistently infect humans: *Plasmodiumfalciparum*, *Plasmodiumvivax*, *Plasmodiummalariae*, and *Plasmodiumovale*. From these, *P.falciparum* and *P.vivax* are the most common with the former being by far the most lethal [2–5]. Recently, different accounts of human malaria by another species, *Plasmodium knowlesi*, which usually infects macaque monkeys, have been reported in Southeast Asia [6].

Since the beginning of the twenty-first Century there has been renewed attention towards the disease, and in 2007 an official research and development agenda for malaria eradication (malERA) was established [7]. The goal is to completely eliminate and, if possible, eradicate the disease from as many areas as possible and control the others. The main strategies being followed include: the increase of insecticidal nets reach, especially long-lasting insecticidal nets (LLINs), within affected communities; use of artemisinin-based combination therapy (ACT) as first-line treatment; and, support the development of a vaccine, with RTS,S/Mosquirix the first being made available and recently approved by the European Medicines Agency (EMA) for children aged 6 weeks to 17 months (exclusively against *P. falciparum*) [8]. Data from 2013 showed that since the beginning of the programme the malaria mortality rate decreased 26 %, even with an increase of 43 % in population living in transmission areas [1]. In the 2015 report, WHO estimates that malaria control interventions averted a total of 663 million malaria cases between 2001 and 2015 in sub-Saharan Africa, which 69 % are accounted to the use of insecticide-treated mosquito nets (ITNs), 21 % due to ACT and 10 % due to indoor residual spraying (IRS) [9].

While these numbers may be interpreted as progress towards winning this war, recent findings revealed some worrying indicators that *Plasmodium* is fighting back. Artemisinin and its derivatives are considered the current last line of defence and have been used in combination with other anti-malarials to avoid resistance development, but artemisinin-resistant strains of *P. falciparum* have appeared in Southeast Asia with strong indications of rapid spread [10–16]. Another worrying indicator is *Anopheles gambiae* (the predominant vector in Africa) resistance to pyrethroids in various sub-Saharan regions [17–20]. The combined proportion of affected population in sub-Saharan Africa with access to IRS and ITNs increased from 2 % in 2000 to 59 % in 2014 [9], and is responsible for reducing child deaths by an average 18 % [1, 21, 22], but also for spreading pyrethroid resistance. There are four different classes that can be used in IRS, but only pyrethroids are currently recommended for LLINs [1, 21].

In recent years, there has been wider interest in mosquito stages as potential targets for new transmission-blocking strategies (TBS) that could help to control, and ultimately, eliminate the disease. New TBS being studied differ mainly from the classical vector control approaches, such as the use of insecticides, because they are designed for mosquito survival, thus avoiding selective pressure towards resistance [23, 24]. Two of the major metrics for malaria transmission intensity are: the basic reproductive number (R_0_) representing the number of new cases deriving from one untreated case in an infinite and susceptible human population; and, the entomological inoculation rate (EIR) that measures the rate of *Plasmodium*-infected mosquito bites per person, per year [25]. Both are of extreme relevance to TBS, not only to provide insights on the impact, but also to set ‘critical numbers’ as goals for a successful strategy [26–30]. Mathematical modelling based on available field data shows that it should be enough to reduce transmission to R_0_ < 1 for a certain period of time to irreversibly compromise the sustainability of the disease [31–34]. In addition, because malaria transmission is a local feature with the source of infection (breeding site) located within a maximum of a 1-km perimeter [29, 35, 36], TBS are particularly suited to circumscribe small pockets of malaria in isolated communities.

There are three main TBS that are being pursued: gametocytocidal drugs, transmission-blocking vaccines and shifting mosquitoes towards refractory traits. Each of these will be briefly reviewed and a short outlook will present bench progress and challenges, especially large-scale implementation and ethical issues, referenced, when possible, with clinical, entomological and field data.

### *Plasmodium* life cycle

Due to the apicomplexan nature of the *Plasmodium* life cycle which allows it to survive in different environments, the parasites are well adapted to their obligatory hosts: a vertebrate and the female of the *Anopheles* genus [37–39].

In humans, the first target of the parasites are the liver cells (hepatocytes), until the point they are released into the blood stream to invade red blood cells in the form of merozoites; it is estimated that each sporozoite that enters the body originates approximately 1000 erythrocyte-infective parasites [40]. Once inside, they reproduce asexually in a (48-h cycle for falciparum) while also forming agglomerates of infected cells to avoid spleen clearance [41]. Eventually, some of the merozoites develop into gametocytes, the sexual form of the parasite, maturing inside the parasitophorous vacuole until released to the peripheral blood, waiting for another mosquito bite to propagate the disease [42]. Parasitaemia in symptomatic infected humans can range from 100 to more than 250,000 parasites per µl of blood (hyperparasitaemia) [43, 44].

**Fig. 1:**
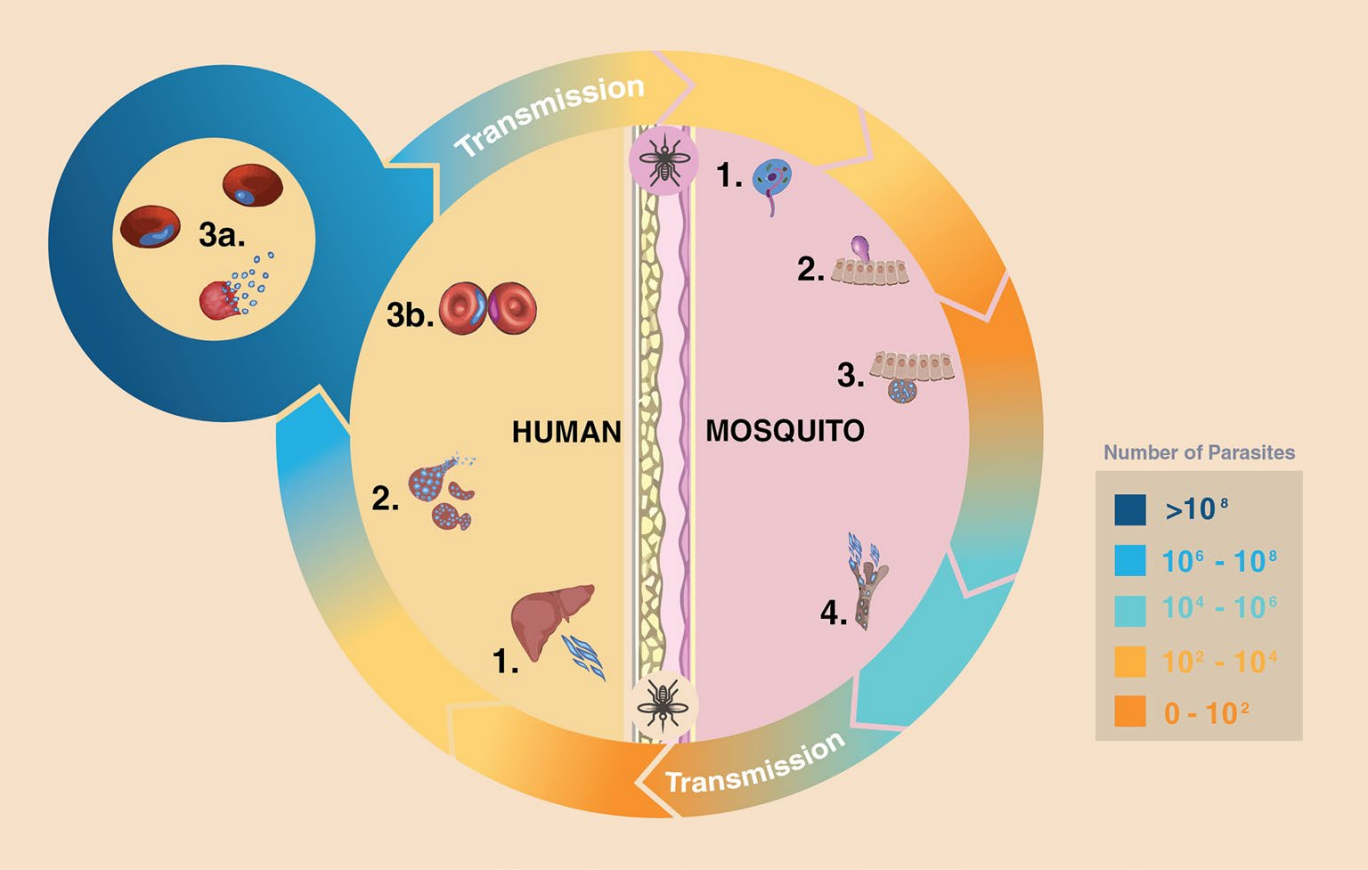
Representation of the malaria life cycle. *Arrows* represent the different stages of infection and parasite density, in humans: *1* invasion of hepatocytes, *2* merozoites maturation and release, *3a* intra-erythrocytic cycle, *3b* gametocytes formation; and, mosquito: *1* gametes egress and fertilization, *2* meiotic division and midgut invasion, *3* oocyst fixation and mitotic division, *4* sporozoites release and salivary glands invasion

When a female *Anopheles* mosquito bites an infected vertebrate host, it results in the ingestion of a certain number of gametocytes. Within 15 min, the midgut lumen environment triggers the gametocyte egress and differentiation into micro and macrogametes [45, 46]. The following fusion leads to diploid cells (zygote) that will undergo meiotic division resulting in motile ookinetes [45, 46]. The main goal of ookinetes is to physically traverse a thick (1–20 µm) chitin-based peritrophic membrane (PM) formed upon blood ingestion, and the midgut epithelium [47–49]. The midgut invasion is not pacific and leads to apoptosis of the invaded cells [50, 51]. After establishing itself at its basal side, a few parasites (less than ten in average) will develop into the oocyst form, concluding one of the most critical stages in whole life cycle (Fig. 1) [45, 46].

Mitotic divisions start occurring at this stage and ultimately sporozoites are formed. Upon egress from mature oocysts, sporozoites travel via the haemolymph and can be found throughout the mosquito haemocoel until some of them reach the salivary glands [45, 46, 52]. Less than 20 % of the sporozoites entering the haemocoel will establish themselves in the salivary gland with the remaining being eliminated [52].

Transmission of sporozoites occurs when the mosquito ejects saliva into the skin, probing for a blood vessel. Despite some mosquitoes can harbour as many as thousands of sporozoites in their salivary glands, only a small number (10–200) are ejected from the salivary ducts [53, 54]. After their injection into the dermis, they will try to find and invade a hepatocyte as soon as possible before detection of the immune system and continue the cycle [55].

In summary, the main differences between the mosquito and humans stages are:

- the mosquito the parasites are exclusively extracellular;
- there are, on average, fewer parasites in the vector comparing to the human host (Fig. 1);
- there are no antibodies produced against *Plasmodium* in the mosquito despite other mechanisms are available to detect and eliminate the parasites [56, 57];
- morphology and proteome of *Plasmodium* differ completely from stage to stage, and the only common cells between hosts (excluding the short period of gametocytes in the mosquito) are the sporozoites [58, 59];
- mosquito infection is not lethal *per se* but can be a metabolic and cellular burden, especially during the midgut crossing stage. If this comes with a fitness cost to the mosquitoes in the field is still not clear, with contradictory claims in the literature [60–62].

### Gametocyte density and prevalence

The proportion of merozoites committed to sexual development and the exact mechanism (including sex ratio determination) are not yet fully understood [63–70]. Gametocytogenesis differs between *Plasmodium* species: while all of them undergo a five-stage maturation process (with some morphological differences), falciparum gametocytes, for example, take the longest (10–12 days as opposed to less than 48 h for the majority) and are not produced simultaneously with the asexual stages. Mature gametocytes persistence in the peripheral blood is estimated to be lower in *P. vivax* (approximately 3 days) than in *P.falciparum* (3.4–6.5 days) [43, 70–72].

High circulating gametocyte numbers do not necessarily result in mosquito infection, and low gametocyte densities do not exclude infectivity; a self-regulated or host-induced response (fever, antibodies, cytokines) still remains unclear [66, 73, 74]. Even so, evidence suggests that transmission success is highly dependent on the proportion of mature gametocytes [75].

Despite all the data available on gametocyte prevalence and density, measuring gametocytaemia levels reliably is still a challenge. The rule of thumb is that the gametocyte count by microscopy techniques usually underestimates the true number by more than 50 % [70, 76]. More sensitive molecular methods such as quantitative nucleic acid sequence-based amplification (QT-NASBA), reverse transcriptase PCR (RT-PCR) and RT loop-mediated isothermal amplification (RT-LAMP) have been developed [77–79]. Despite their usefulness, there is the obvious cost and training barriers for applying any of these technologies in the field when the available resources, both human and capital, are already scarce. Thus, a thorough analysis from local authorities and health policy makers to invest in the right resources should be made, being it microscopy, rapid diagnosis tests (RDT) or more advanced molecular biology tools (or a combination of all), resulting in a clear diagnosis strategy defined according to: epidemiological settings of prevalence and density; the health impact of the disease using metrics like the disability-adjusted life year (DALY) in the local population; and economical, not only immediate, such as throughput and cost per unit/case and treatment costs avoided, but also long term, factoring the costs of morbidity and mortality in the socioeconomic advancement of the affected society [80–83].

Various studies have also attempted to estimate the relative contribution to mosquito infection of different patient age groups [68, 84–88]. Despite some evident differences in gametocyte density and prevalence between adults and children, the data are not conclusive and are hard to compare due to lack of consistency in gametocytes estimation methods but also in infection models (skin or membrane feeding assays) [29, 89]. The non-linear relation, especially at low densities, between human parasitaemia and mosquito infectiousness, and differences in the endemic settings further enhances the challenge in drawing conclusions from these studies. A thorough understanding of the factors determining transmission would not only allow mathematical models to predict the relative contribution of different groups to transmission, but also help to find the most efficient way to implement a TBS and measure its impact.

### Transmission-blocking strategies

#### Gametocytocidal drugs

One of the first approaches to be explored was the gametocytocidal activity of commercially available anti-malarial drugs [90]. The rationale was to block transmission by clearing most gametocytes in the human host to render those patients non-infectious to mosquitoes. ACT (artemisinin-piperaquine) combined with a low dose of primaquine (PQ) has been used to help eliminate malaria from 17 Cambodian villages [91], although it is hard to make a clear distinction between the direct effect on gametocytes clearance and the indirect one of ACT reducing the numbers of asexual parasites that could develop into gametocytes [92]. Nevertheless, recognizing the importance of reducing transmission, in 2010 WHO recommended a single dose of PQ of 0.75 mg/kg as a gametocytocidal agent (not sufficient for hypnozoite clearance in *P. vivax* infections), in combination with ACT making it the first TBS to be endorsed for field application. However, this dose level has restrictions as it should not be given to pregnant women or small children and because there is the risk of haemolysis in people affected with glucose-6-phosphate dehydrogenase (G6PD) deficiency, although it most be said that the deficiency is originated from different variants that translate into different phenotypes (from mild to severe) that must be accounted when determining PQ safety in such patients [93–95]. Finally, in 2012, WHO revised their recommendation to 0.25 mg/kg in a single dose, but this is still not advised for pregnant women and infants [96].

Responsiveness to drugs is something difficult to compare between *Plasmodium* species. For *P. vivax* only a few clinical data are available because continuous ex vivo culture methods are not yet ready to screen drugs for gametocytocidal activity [70, 97–102]. Even with in vitro culture, where different protocols of sexual stages of *P. falciparum* from gametocytes to ookinetes have been recently made available [103, 104], a relevant limitation in screening anti-malarials and new molecular entities (NMEs) for gametocytocidal activity is the throughput of such models. It certainly opened the door for a wide variety of drugs to be tested using different methods such as ATP bioluminescence, confocal fluorescence and AlarmBlue oxireduction [105–109], but the variability between activity measurements of the same drug, sometimes even with the same method (Table 1), and the lack of a true high-throughput model that is able to screen more than 1000 molecules at a time to effectively search for leads and new scaffolds to design the best drugs for these stages, indicates there is still a lot of progress to be made. There have been some efforts and advances in this direction [110–112] but still far from the standard screening models available for asexual stages [113].

**Table 1:** Comparison of in vitro gametocytocidal activities with different methods and anti-malarial drugs

Drug	IC50 uM (stage IV–V)	Method	References
Primaquine	>10	ATP bioluminescence	[107]
	7.2	HTS confocal fluorescence	[109]
Mefloquine	100	HTS confocal fluorescence	[109]
	4.7	ATP bioluminescence	[107]
Pyronaridine	4.260	HTS confocal fluorescence	[109]
	3.2	ATP bioluminescence	[107]
	0.28	ATP bioluminescence	[106]
Pentamidine	0.404	ATP bioluminescence	[106]
	2.85	HTS confocal fluorescence	[109]
Methylene Blue	0.287	HTS confocal fluorescence	[109]
	0.49	ATP bioluminescence	[107]
	0.012	ATP bioluminescence	[106]
Diihydroartemisin	0.00217	HTS confocal fluorescence	[109]
	3.56	ATP bioluminescence	[107]
Artesunate	2.53	HTS confocal fluorescence	[109]
	2.3	ATP bioluminescence	[106]
	10.48	ATP bioluminescence	[107]
Epoximicin	0.00042	ATP bioluminescence	[107]
	0.0014	Alamar Blue	[108]
	0.00066	HTS confocal fluorescence	[109]

Most of the research is focused on the late gametocyte stage but it is also possible to target the mosquito stages of the parasites (sporontocidal activity), as demonstrated with proguanil, pyrimethamine and most endoperoxides [70, 105]. The rate and timing at which gametocytes are cleared or neutralized (transmission time-window) is a factor to take into account when considering a transmission blocking drug, for example a single dose of PQ that has very short-live and targets only late gametocytes, may leave a certain number of gametocytes behind that will eventually mature and able to infect mosquitoes [114–116]. This would not be the case if used in combination with a sporontocidal drug, but on the other hand the long half-life needed to be effective, is a major challenge to overcome.

A theoretical model recently raised concern by predicting a negative impact on drug resistance for gametocytocidal campaigns, by potentially reducing the transmission of drug-sensitive sexual forms to a greater extent than the drug-resistant ones [117]. Despite the fact that some data suggest resistance is spreading faster at both ends of the transmission-intensity spectrum [117–120], this possibility has not been conclusively confirmed or refuted by empirical data and, probably, the implications for superinfection (simultaneous infection with multiple genetically distinct parasites) should also be accounted [121].

Another model suggested that increasing ACT coverage would outperform the addition of any specific gametocytocidal drug in reducing transmission [122], but even if true, the spreading of artemisinin resistance and PQ side effects should suffice for investing time and resources in the development of a novel, efficacious, late-gametocyte/gamete inhibitor.

### Transmission-blocking vaccine

During 2013, the Malaria Vaccine Initiative (MVI) updated its roadmap and included the development of vaccines interrupting malaria parasite transmission (VIMTs) as one of its strategic goals for the next years [123, 124]. The vaccine approach aims to achieve transmission reduction through an immunological attack on sexual or mosquito stages of the life cycle. To this end, an immune response in the human host directed to stage-specific targets is required.

Surface proteins of gametocytes and gametes (Pfs 2400, Pfs 230, Pfs 48/45, Pfg 27), zygote and ookinete stages (Ps 25, Ps 28) have been the principal candidates [70, 125–128], but other epitopes from later stages (Ps 21), molecules such as chitinase, which is important for PM and midgut traversal, and alanyl aminopeptidase (AnAPN1), an antigen in the midgut surface, important for ookinete recognition, were identified as potential targets [129, 130]. Since there is a population chokepoint occurring, the appeal, in terms of resistance spread and elimination purposes, of targeting later stages in the mosquito infection is obvious, even if the quantitative translation of reducing parasite numbers in different stages into a successful transmission and propagation of malaria is not well enough understood [45, 46, 124, 131].

To develop a successful vaccine, choosing the best target is just the first step, and the need to find a good production and delivery system, complemented by an appropriate formulation and potential adjuvants, is also important [132, 133]. Apart from killed or attenuated whole pathogens (non-practical for mosquito stages) [134, 135], large-scale production of protein antigens is doable using various recent technologies, either synthetic or recombinant, but correct folding, a prerequisite for achieving highly specific high titres in humans is still a challenge [136–138]. In the case of malaria and transmission-blocking vaccines (TBVs) in particular, several expression systems for recombinant antigens have been used, including *Escherichia coli*, *Lactococcus lactis* bacterium models, *Baculovirus*, yeast (*Pichia pastoris, Saccharomyces cerevisae)*, plant-based systems, and algae [138–144]. Particle-delivery technology, such as virus-like particles (VLP) and nanoparticles, are also being pursued [143, 145], and the recently developed DNA vaccine technology has been tried but it is still in a very early stage of development [146–148].

Some candidates using different antigens, production and delivery strategies are currently in preclinical stage and being considered for the first tests in humans [149], and others, such as AnAPN1, are still at earlier stage but nevertheless promising because the antibodies produced appear to inhibit both *P. falciparum* and *P. vivax* [129]. Targeting *P. vivax* is of particular importance since this parasite can stay undetected in the liver in the hypnozoite dormant over 2 years, with PQ being the only medicine to target them at the moment [150]. Two different recombinant strategies (using virus-like and conjugating with *Pseudomonas aeruginosa* exoprotein A) targeting Pfs25 antigen are already in phase I of clinical trials [151, 152], and are the first official candidates for VIMTs targeting sexual, sporogonic or mosquito-stage antigens (SSM-VIMT) but are still a small fraction of the malaria vaccine pipeline [153].

In addition to the development of a stand-alone SSM-VIMT, which would not confer immediate benefit to a vaccine recipient, a vaccine targeting both SSM and other stage-malaria antigens is being studied. Examples of such an approach are the fusion of circumsporozoite protein (CSP), Pfs 25 and glutamate-rich protein (GLURP) with Pfs 48/45 [154–156]. Just as targeting antigens from multiple parasite stages may create synergies, the use of a vaccine and drug together could also maximize the impact on transmission for a longer period than a drug alone could. It was also suggested that a SSM-VIMT could be coformulated or co-administrated with another health intervention (as Mosquirix with hepatitis B) that targets the same population to render it directly beneficial to recipients, and overcome some of the ethical issues associated with this kind of intervention [157, 158].

### Refractory mosquitoes

Mosquitoes have their own natural mechanisms to fight *Plasmodium*, with a small percentage of them being refractory to the infection [38, 56, 57]. Researchers have recently started to explore possible ways to exploit this observation in the malaria elimination effort. The idea is to (naturally or artificially) emulate the refractoriness processes in the laboratory and somehow introduce it in the field, in such a way that will eventually shift the mosquito population towards a *Plasmodium*-resistant vector phenotype. Some of the strategies were borrowed from other vectors and disease control programmes and are already tested in the field on a small scale, and others still are in an early stage of research [158–161]. They fall into three categories: population replacement, artificial gene drive mechanisms and third-party modified organisms as delivery systems. Their common feature is the expression of at least one effector molecule responsible for malaria refractoriness. Since this requisite is not met with population suppression methods, such as the release of insects carrying a dominant lethal genetic systems (RIDL), it is not included in this topic.

An ideal effector molecule will not convey a fitness cost to the insect host and could be used in combination to target different stages of the parasite development. Some candidates already tested include phospholipase A2 (PLA2), shown to inhibit ookinete invasion through an unknown mode of action [162], and salivary gland and midgut peptide 1 (SM1) that is thought to block recognition sites for sporozoites and ookinetes [163].

Population replacement can be achieved by releasing either genetically modified (GM) mosquitoes that express a killing or disabling agent for the malaria parasites [164], or naturally refractory mosquitoes previously selected and reared in the laboratory [162]. For large-scale implementation in the field, mosquito releases would probably need to be preceded with intensive insect elimination campaigns to reduce the native population. Release should be done in a controlled manner backed up with a mathematical model giving insight for the stage, numbers, timing, and location that would maximize the desired genetic shifting of the population [165]. Even if the fitness cost of the desired trait is designed to be minimal, it is very likely that this shifting will not be incremental and self-sustainable, and in order to maintain the modifications, periodic releases of additional insects might be needed [161]. Not only is it difficult and costly to rear such large numbers of mosquitoes to have a meaningful impact on levels of transmission of the disease, there is also the ethical issue behind releasing biting female *Anopheles*, and before being implemented, a careful sensibilization campaign should be made within affected communities.

As an alternative strategy, gene drive mechanisms have been explored to reduce the number of insects needed (males only) to propagate in a self-sustainable manner without additional releases [161, 164]. Many solutions have been studied, starting from transposable elements that will be integrated randomly across the genome, or ‘selfish genes’, such as the homing endonuclease genes (HEGs) that are transmitted horizontally within a population by using the host cell DNA repair machinery. The linkage between the drive mechanism and the refractory system deserves special attention and must be engineered in a way that, in case it is broken, there is a safety system to prevent modified insects overtaking the population [161, 164]. A system consisting of synthetic genetic elements, with mosquito regulatory regions and the HEG I-SceI11-13, has already been demonstrated to substantially increase its transmission to the progeny of *An. gambiae* [166], and very recently a Cas9-mediated gene drive mechanism was published with very interesting results [167–169].

Finally, life organisms, such as bacteria and fungi [170–172], and even viruses [173], have been proposed to be engineered as expressing systems for refractory genes inside the mosquitoes. The diversity of the midgut flora in adult *Anopheles* is well known and includes *Escherichia, Alcaligenes, Pseudomonas, Serratia*, and *Bacillus*, which could be used as paratransgenesis vehicles [174]. One such system has been demonstrated with *Escherichia coli* expressing a fusion protein of ricin and a single-chain antibody against Pbs2, inhibiting oocyst formation by 95 % in *Anopheles stephensi* [175]. The bacterium *Wolbachia* is a special case that has received much attention lately, as it was observed that infected *Anopheles* became refractory to malaria to a significant extent without the need of further genetic manipulation, although the detailed molecular mechanisms are still unclear*. Wolbachia* inserts a drive mechanism through cytoplasmic incompatibility, meaning that only when both female and male are infected, the fertilized eggs will hatch normally and the bacteria is transovarially transmitted to the next generation [160, 161, 176]. Another possibility is the use of entomopathogenic fungi: fungal spores may be extremely robust and have the advantage to infect mosquitoes directly via the cuticle. Fungal spores can be integrated into a number of delivery systems, such as indoor spraying or odour-baited traps [159, 177, 178]. One such example is *Metarhizium anisopliae*, modified to express a fusion protein between SM1 and scorpine (antimicrobial toxin), which was able to reduce *P. falciparum* sporozoite counts by 98 % in *An. gambiae* [178].

### Future perspectives

A successful wide-scale implementation of TBS for malaria elimination campaigns is still some way in the future. Each strategy has its own unique challenges but there are some common questions and technical limitations that need to be overcome and answered. One of the central questions in TBS research is the quantitative and qualitative relationship between transmission reduction and its impact in the parasite reservoir. Several studies have been made [45, 46, 69, 84–88, 124, 131], but there is still a huge knowledge gap to be filled. Better data are needed for human-to-mosquito (and vice versa) transmission rates, prevalence and density of different mosquito stages, and the relation with geographic and demographics (both human and mosquito). The current limited availability of such data is both a challenge and an avenue for further research to translate TBS from the laboratory bench to the field.

Testing the efficacy of a future transmission-blocking drug or vaccine is a further important requirement. Randomized cluster approaches can be used but the endpoints to assess the clinical relevance of such therapeutics are still not clear [137, 157, 179]. The ideal endpoint should be the reduction of new human infections from the same epidemiological source following the introduction of the intervention, but is very difficult to achieve in the field. An alternative is to measure the reduction in the total number of cases, but then it is impossible to assess the direct contribution of the treatment. Whichever endpoint is chosen, the real world will require a compromise between directly quantifiable impact and epidemiological relevance.

A major concern of TBS is regulatory approval, because a clinical development plan for TBS will certainly be different from those applied to other malaria vaccines and drugs. Encouraged by the US Food and Drug Administration (FDA) which clearly states that there is no legal opposition to licensing, and in view of the momentum of new SSM-TBV projects reaching clinical testing, a work group of international experts was established to assess the requirements of an eventual SSM-VIMT Phase III trial [179]. One of the conclusions highlighted the critical importance of identifying the minimally required efficacy (and coverage) and the need for specific criteria that will inform early clinical decision-making.

Both, compartmental and mechanistic mathematical modelling, are paramount for the progress of any of the strategies discussed above [180–182]. This was also one of the lessons learned during the previous Global Malaria Eradication Programme (GMEP) during the 1950s and 1960s, where dichlorodiphenyltrichloroethane (DDT) was widely used as insecticide until the programme was discontinued due to a combination of resistance, financial constraints and negative environmental impact [183]. Some studies suggest that despite the immediate successes of the programme in certain regions, in others, the gain was lost with resurgence appearing in endemic proportions [184]. Thus, the aim should be a long-term strategy, able to measure and predict its impact locally in a specific set of conditions. This will then allow informed decisions on how, where, and when is the best way to implement a certain TBS, and good models and data are crucial to reach that goal [185, 186].

**Fig. 2:**
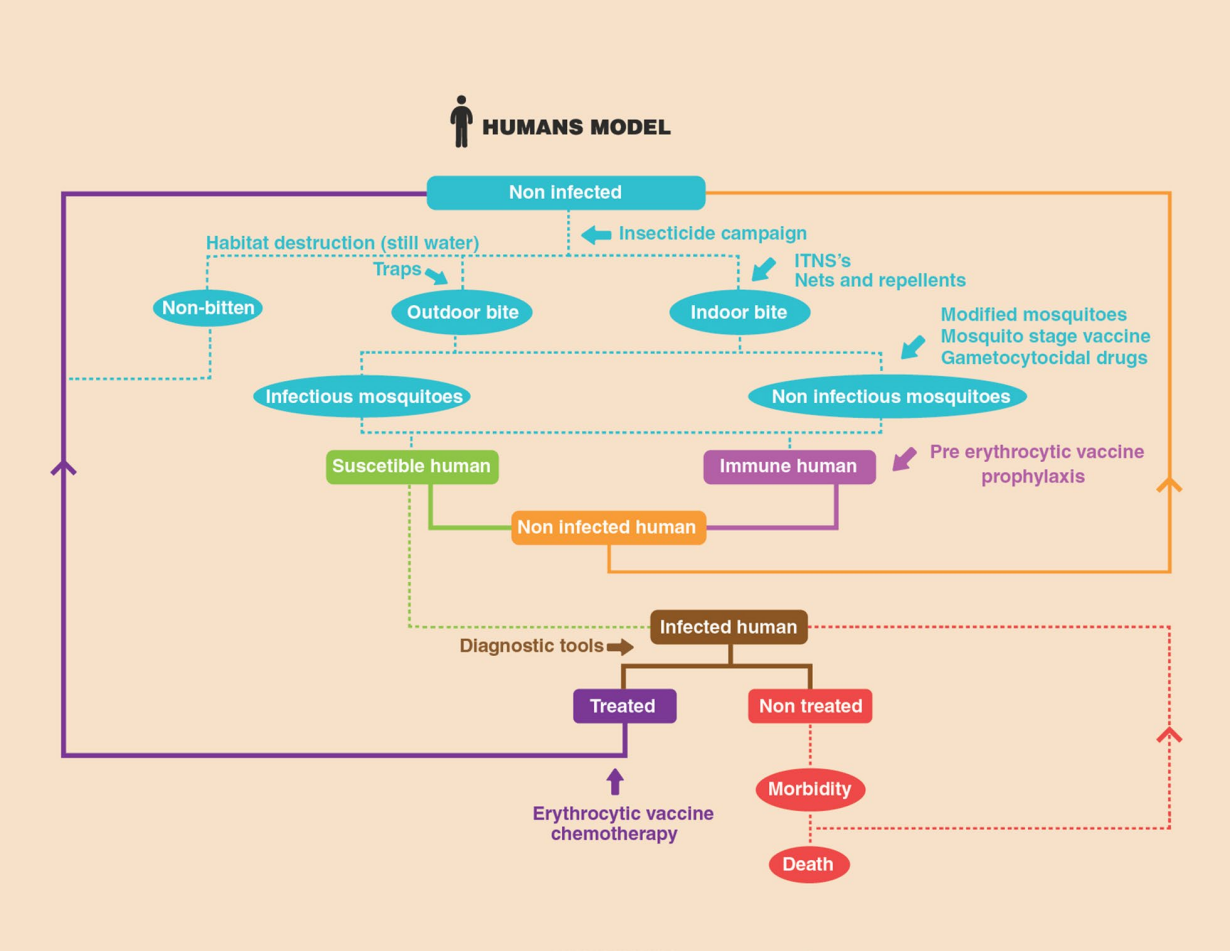
Schematic model of malaria infection and possible outcomes from the human perspective. *Highlighting* potential points of intervention for different strategies for malaria eradication available or under study

**Fig. 3:**
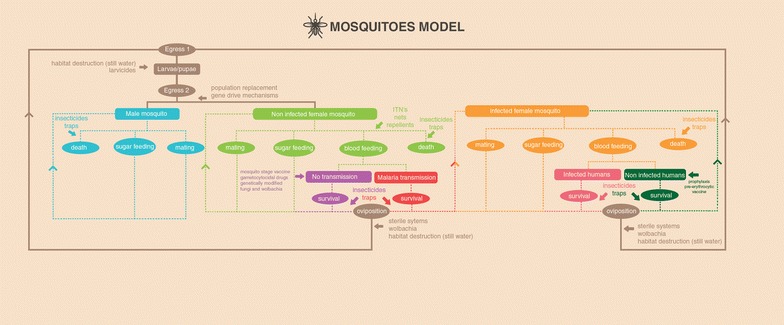
Schematic model of malaria infection and possible outcomes from the mosquito perspective. *Highlighting* potential points of intervention for different strategies for malaria eradication available or under study

Even if all these challenges are overcome, the cost-benefit issue remains. Being a disease that targets mainly populations of underdeveloped countries, cost is always a primary challenge for any type of intervention [187]. Not only the benefit should be proven, but the cost-per-unit should be optimized to make it affordable for mass campaigns. Logistics is another constraint usually associated with malaria since most affected populations live in very remote regions. A positive indicator came from a recently developed vaccine against meningitis (MenAfriVac^®^), costing less than US$0.50 per dose and able to be removed from constant refrigeration, making it more accessible for handling in remote areas of sub-Saharan Africa [157, 188]. Such features would be highly desirable for a malaria vaccine, but the central question still remains: is it really worth allocating that many resources to develop and implement TBV campaigns in less than optimal efficacy and coverage conditions as seen with Mosquirix [189, 190]? If used in combination with other tools, the answer might be affirmative, the main focus is not to have the maximum clinical effect but to strike a precise and coordinated blow to the parasite reservoir (human and mosquito) to reach a minimum threshold that will compromise its propagation and eventual survival in focal, and eventually global, areas (Figs. 2, 3).

Finally, there is the ethical issue behind using medicines without direct therapeutic benefit to patients, even considering the decreased probability of re-infection. Even if there were no direct benefits, one can argue that individuals taking TB medicines or vaccines is a matter of public health. Education and population sensibilization prior implementation, maybe even more than cost and efficacy, is therefore crucial for any strategy to succeed.

On the other hand, all strategies presented in the ‘refractory mosquitoes’ topic are far from implementation; despite some promising results, more knowledge such as the epidemiological effects of releasing modified mosquitoes and biopathogens in the field, is needed [164, 191]. Such unconventional approaches might disfavour public awareness and support, but if enough evidence of the positive impact for malaria elimination is gathered and its scalability is proven, then they could become another weapon in the TBS arsenal.

## Conclusions

While it is not expected that TBS will be sufficient to eliminate the disease on their own, their application has the potential to boost other strategies. TBS should be aimed to the lowest endemicity level areas possible (R_0_ < 10) with frequency and timepoints optimized, taking into account not only the immediate impact in the human reservoir of parasites, but also the long term. In addition, efforts to find the best solution for endpoints and regulatory challenges at clinical trials still need to be made and sensibilization campaigns in affected communities to educate about the beneficial issues of TBS should be a priority.

Any future conventional vaccine for human stages of the disease most likely will be a compromise between efficacy, long-lasting immunity and population coverage. Even if new drugs and insecticides come to market, or resistance against ACT is contained, it is a matter of time until they are rendered ineffective. To achieve malaria elimination, all stages should be targeted, preferably at once, and some of the TBS described here, or others to come, most certainly will have a major role to play.
